# Active sensing explains cortical variability and improves visual information

**DOI:** 10.64898/2026.10.12.758571

**Published:** 2026-10-19

**Authors:** Ryan A. Ressmeyer, Declan P. Rowley, Tejas Kothapalli, Dekel Galor, Ruei-Jr Wu, Michele Rucci, Jude F. Mitchell, Daniel Butts, Alexander C. Huk, Jacob L. Yates

## Abstract

Active vision is often considered at the scale of saccades, with each fixation treated as a static visual sample. Yet the eyes continue to move during fixation, continually transforming the retinal input. Here we measured these movements at high precision while recording neuronal populations in the foveal primary visual cortex of awake marmosets viewing repeated flashed natural images. Fixational eye movements accounted for most conditional firing-rate variability and nearly all shared variability not explained by the trial-averaged response. An image-computable model captured this modulation largely from gaze-dependent retinal input; explicit eye-position and eye-velocity signals added little predictive power. Under natural viewing, retinal motion redistributed image power across temporal frequencies, and motion effects grew when this power engaged spatiotemporal tuning. In model-derived populations, fixational motion increased the spatial information carried per spike relative to stabilized input, with sharpening growing across successive nonlinear stages. Thus, activity commonly assigned to cortical noise is not just structured visual reafference generated by the movement of the observer, but is beneficial signal which sharpens spatial representations.

